# Supplementary figures and images for: Genetic Reconstruction and Forensic Analysis of Chinese Shandong and Yunnan Han Populations by Co-Analyzing Y Chromosomal STRs and SNPs
Source: Genes (Basel). 2020 Jul 3;11(7):743. doi: 10.3390/genes11070743 (PMC7397191; doi:10.3390/genes11070743)

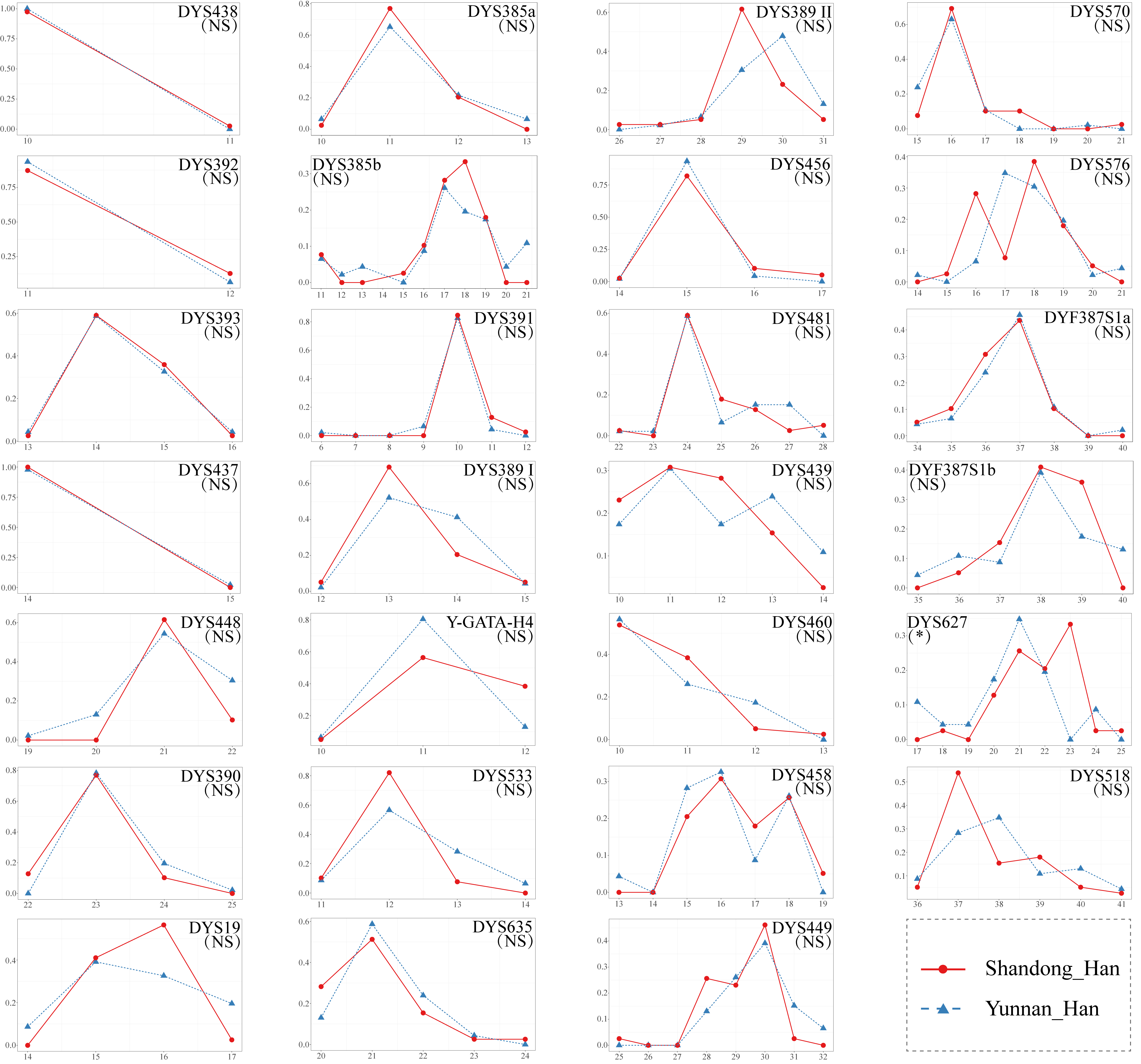

Supplement: Supplementary file 1 [file genes-11-00743-s001.zip › supplementary files/Figure S1.tif]

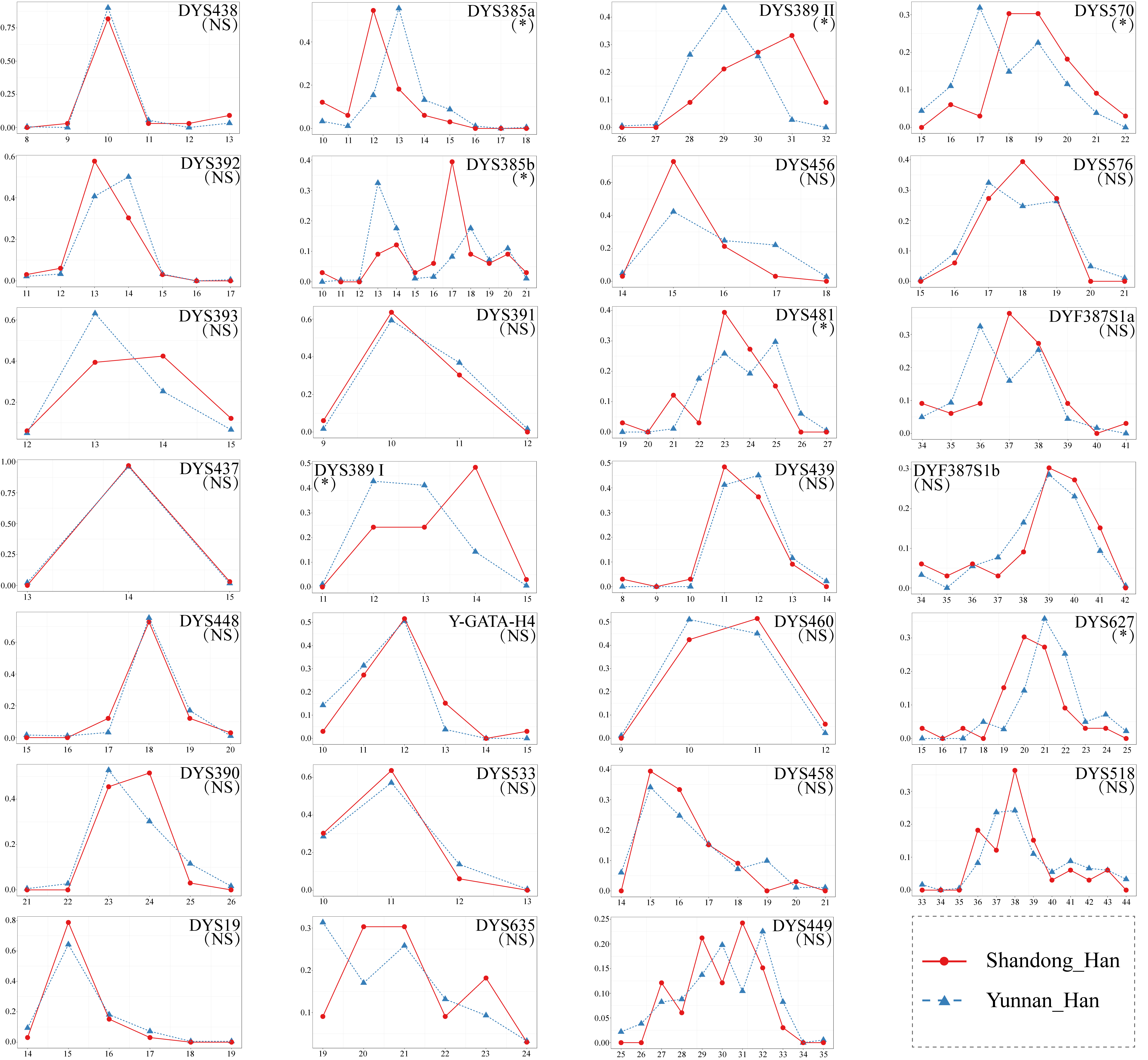

Supplement: Supplementary file 1 [file genes-11-00743-s001.zip › supplementary files/Figure S2.tif]

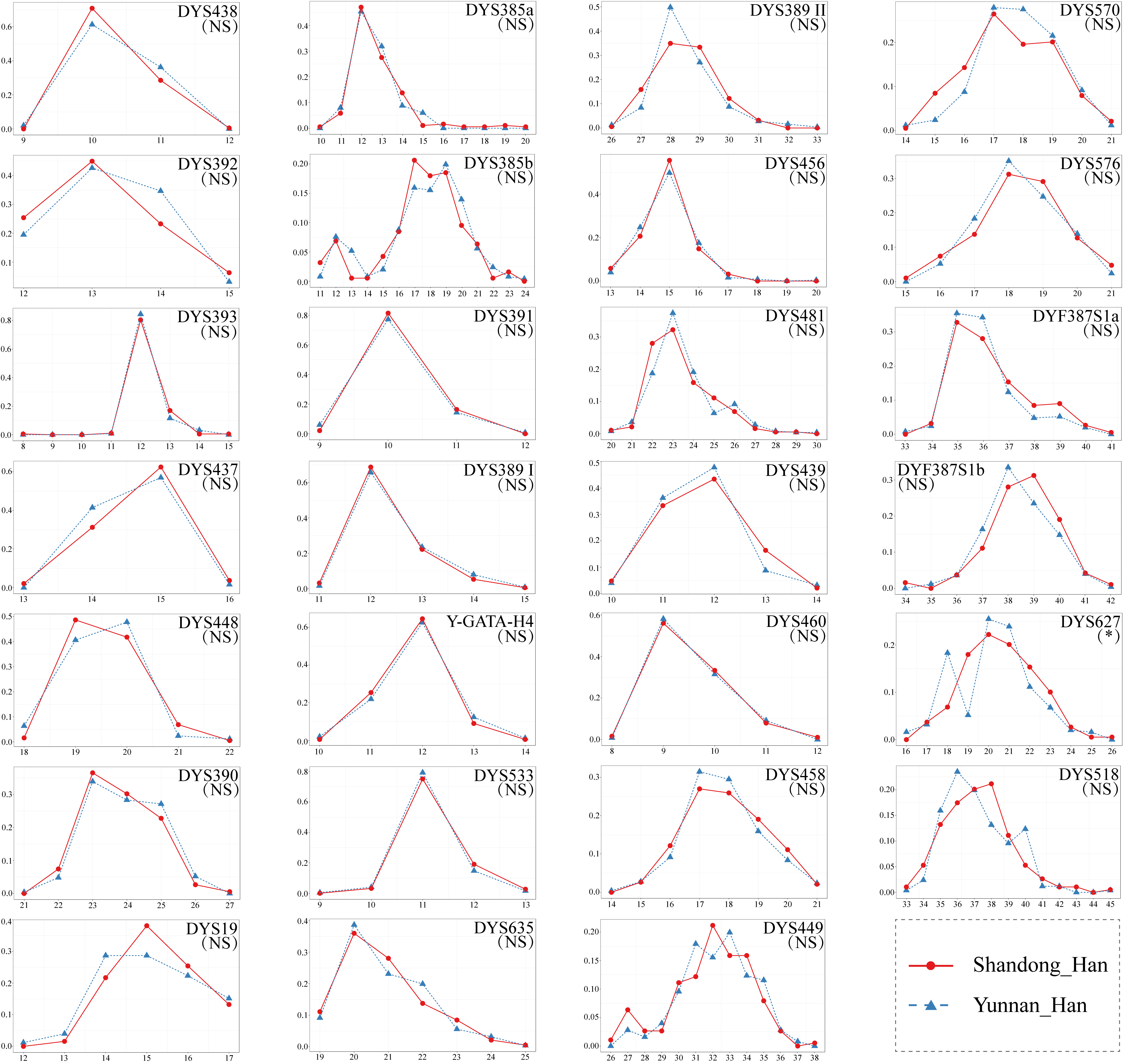

Supplement: Supplementary file 1 [file genes-11-00743-s001.zip › supplementary files/Figure S3.tif]
